# Unbiased transcriptomic analyses reveal distinct effects of immune deficiency in CNS function with and without injury

**DOI:** 10.1007/s13238-018-0559-y

**Published:** 2018-06-28

**Authors:** Dandan Luo, Weihong Ge, Xiao Hu, Chen Li, Chia-Ming Lee, Liqiang Zhou, Zhourui Wu, Juehua Yu, Sheng Lin, Jing Yu, Wei Xu, Lei Chen, Chong Zhang, Kun Jiang, Xingfei Zhu, Haotian Li, Xinpei Gao, Yanan Geng, Bo Jing, Zhen Wang, Changhong Zheng, Rongrong Zhu, Qiao Yan, Quan Lin, Keqiang Ye, Yi E. Sun, Liming Cheng

**Affiliations:** 1 Division of Spine Surgery, Department of Orthopedics, Tongji Hospital, Tongji University School of Medicine, Shanghai, 200065 China; 2 Institute of Spine and Spine Cord Injury of Tongji University, Shanghai, 200065 China; 3 Stem Cell Translational Research Center, Tongji Hospital, Tongji University School of Medicine, Shanghai, 200065 China; 4 Department of Psychiatry and Biobehavioral Sciences, David Geffen School of Medicine, University of California Los Angeles, Los Angeles, CA 90095 USA; 5 Department of Pathology and Laboratory Medicine, Center for neurodegeneration disease, Emory University School of Medicine, Atlanta, GA 30322 USA

**Keywords:** spinal cord injury repair, immune deficiency, transcriptomic analysis, neurotransmision

## Abstract

The mammalian central nervous system (CNS) is considered an immune privileged system as it is separated from the periphery by the blood brain barrier (BBB). Yet, immune functions have been postulated to heavily influence the functional state of the CNS, especially after injury or during neurodegeneration. There is controversy regarding whether adaptive immune responses are beneficial or detrimental to CNS injury repair. In this study, we utilized immunocompromised SCID mice and subjected them to spinal cord injury (SCI). We analyzed motor function, electrophysiology, histochemistry, and performed unbiased RNA-sequencing. SCID mice displayed improved CNS functional recovery compared to WT mice after SCI. Weighted gene-coexpression network analysis (WGCNA) of spinal cord transcriptomes revealed that SCID mice had reduced expression of immune function-related genes and heightened expression of neural transmission-related genes after SCI, which was confirmed by immunohistochemical analysis and was consistent with better functional recovery. Transcriptomic analyses also indicated heightened expression of neurotransmission-related genes before injury in SCID mice, suggesting that a steady state of immune-deficiency potentially led to CNS hyper-connectivity. Consequently, SCID mice without injury demonstrated worse performance in Morris water maze test. Taken together, not only reduced inflammation after injury but also dampened steady-state immune function without injury heightened the neurotransmission program, resulting in better or worse behavioral outcomes respectively. This study revealed the intricate relationship between immune and nervous systems, raising the possibility for therapeutic manipulation of neural function via immune modulation.

**Electronic supplementary material:**

The online version of this article (10.1007/s13238-018-0559-y) contains supplementary material, which is available to authorized users.

## Introduction

The mammalian central nervous system (CNS) is generally considered incapable of regeneration. Spinal cord injury (SCI) often results in permanent loss of movement, sensation and autonomic function to varying degrees depending on the position (e.g., along the anteroposterior axis) and the nature of the injury. To date, SCI is still considered as an incurable disease. While standard medical and surgical care with additional rehabilitation procedures have been implemented in SCI treatment, more effective interventions for spinal cord repair are still sought by many scientists and clinicians (McDonald and Sadowsky, 2002; Lu et al., 2012; van den Brand et al., 2012).

One of the pathological hallmarks of traumatic SCI in mammals is the activation of both residential and infiltrating immune cells accompanied by the release of chemokines and cytokines, which participate in both acute and chronic host defense responses (Popovich and Jones, 2003). Despite the prevailing notion that inflammatory immune responses are the major cause of secondary lesions after SCI, i.e., further damage to the nervous system beyond the initial traumatic impact, whether immune defense responses play a beneficial or detrimental role in the entire course of SCI progression is heavily debated (Popovich and Longbrake, 2008). Given that boosting immunity could lead to visible increases in neuronal and glial cell death, the immune-boosting strategy is currently facing challenges in the clinic for SCI repair (Popovich and Longbrake, 2008).

Our previous work had demonstrated that neutrophin-3 (NT-3)-loaded chitosan provides an excellent microenvironment, which is anti-inflammatory, neurogenic, neurotrophic, and angiogenic, for SCI repair and functional recovery in a rat model of SCI (Duan et al., 2015; Yang et al., 2015). Using severe combined immune deficiency (SCID) mice, we and others provided initial evidence that immune deficiency was linked to better functional recovery after SCI (Duan et al., 2015; Luchetti et al., 2010; Yamanaka et al., 2016; Kigerl et al., 2006). SCID mice, which are often used as a model for cell transplantation studies, carry spontaneously-arising recessive loss-of-function mutations in the *Prkdc* gene encoding protein kinase, DNA activated, catalytic polypeptide (PRKDC), on chromosome 16. PRKDC is an enzyme involved in DNA repair. SCID mice lack V(D)J recombination thus the humoral and cellular immune systems fail to mature (Bosma et al., 1983). SCID mice do not have mature T cells or B cells and are incapable of mounting proper adaptive immune responses. To further study the intricate relationship between adaptive immune responses and neuronal function after SCI as well as the underlying molecular mechanisms, we carried out “big-data” based, unbiased transcriptomic analyses of spinal cord segments before and after crush injury in wild type and SCID mice. Weighted gene coexpression network analyses (WGCNA) revealed that dampened immune functions were linked to elevated neural transmission program, leading to better neural functional recovery.

Interestingly, objective transcriptomic analyses revealed an unexpected event, i.e., in uninjured spinal cord of SCID mice, neurotransmission-related genes had heightened expression, indicative of potential neuronal hyper-connectivity. Historically, the CNS has been considered as an immune privileged organ in mammals due to the presence of the blood-brain-barrier (BBB), which was thought to arise from an evolutionary adaption to restrict immune responses within the CNS (Streilein, 1995). However, recent studies indicate that lymphatic vessels (Iliff et al., 2015; Louveau et al., 2015) as well as memory T cells present in CNS meninges (Kivisäkk et al., 2003) are important for immune surveillance, neurogenesis, spatial learning, neuronal connectivity and social behavior (Filiano et al., 2017). Moreover, a number of mental disorders including schizophrenia, autism and Rett syndrome, are known to be characterized not only by abnormal behaviors and worsened cognitive performance, but also by immune dysfunction (Richard and Brahm, 2012; Michel et al., 2012). Our transcriptomic analyses suggested that deficiencies in immune responses are tightly linked to elevated neural transmission, or potentially, to neuronal hyper-connectivity, which could lead to abnormal cognitive behavioral outcomes.

Our study reveals an intricate relationship between immune responses and neuronal functions. Such a relationship explains how dampened immune responses in injured spinal cord result in better neural functional recovery. It also elucidates how immune deficiency, in the absence of injury, leads to diminished learning probably through neuronal hyper-connectivity.

## Results

### SCID mice demonstrated better functional recovery after SCI

**Figure 1:**
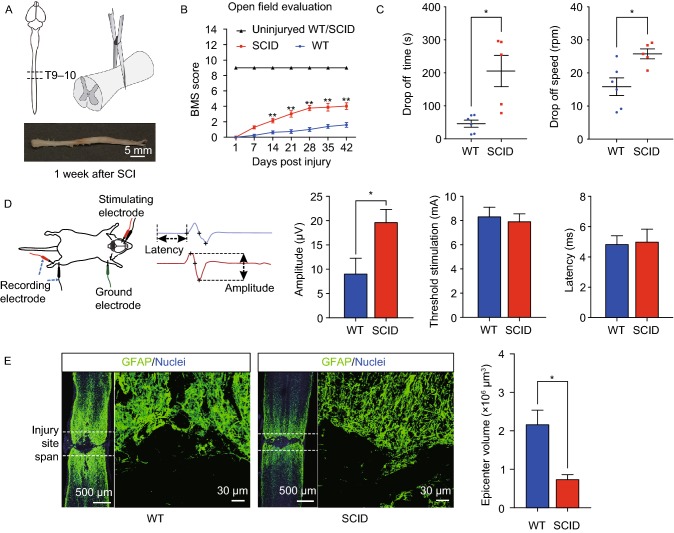
**SCID mice showed better motor functional recovery than WT mice after SCI**. (A) Schematic illustration of anatomical positioning of SCI (upper left diagram) and SCI crush model using an artery clamp with 60 g constant force (upper right diagram). The lower panel shows an example of the gross anatomy of spinal cord tissue 1 week post-injury. (B) BMS open-field test to show hindlimb motor functional recovery of SCID and WT mice from 0 d to 42 d after SCI (mean ± SEM; **P < 0.01, *n* ≥ 15, unpaired Student’s *t*-test with comparison between two groups at each time point). (C) Rotarod performance of WT and SCID mice at 42 d post-injury. The left panel shows the drop-off time at 15 rpm constant speed, and the right panel shows the drop-off speed during 0 to 40 rpm acceleration (mean ± SEM; *n* = 6, *P < 0.05, unpaired Student’s *t*-test). (D) Electrophysiological analysis of SCID and WT mice at 42 d post-injury. The left two panels show a schematic graph of MEP recording and representative MEP traces. The right three panels show significant differences in Amplitude (Amp), but not in threshold stimulation (TS) or latency between SCID and WT groups. (mean ± SEM; *n* ≥ 5, *P < 0.05, unpaired Student’s *t*-test). (E) GFAP immunohistochemical analysis demonstrated significantly greater lesion volumns in WT mice than SCID mice. Dotted lines demarcate the epicenter span with high magnification images to the right of each panel (mean ± SEM; *n* = 6, *P < 0.05, unpaired Student’s *t*-test)

Both SCID and wild-type (WT) mice were subjected to SCI at T9–10 with a modified bulldog clamp for 3 s, which provided a constant force of 60 g (Fig. 1A). Gross locomotion was assessed using the basso mouse scale (BMS) open-field locomotor test before injury, and 1, 7, 14, 21, 28, 35, and 42 d after injury (Fig. 1B). SCID mice demonstrated significantly better hindlimb recovery compared to WT mice from 14 d post-injury and onward, achieving a final score of 4. In contrast, WT mice had moderately increased BMS scores at 14 d post-injury, but scores remained lower than 2 for up to 42 d post-injury (Fig. 1B). We evaluated motor coordination using a rotarod at 42 d post-injury. In this test, SCID mice showed better performance as measured by higher drop-off speed during 0 to 40 rpm acceleration and longer time to drop-off at a 15 rpm constant speed (Fig. 1C). These results indicated better walking balance and coordination in SCID mice after SCI. We subsequently carried out motor evoked potential (MEP) recording to assess the integrity of the motor pathway and functional recovery. The peak-to-peak amplitudes were calculated as amplitude values of MEP (Amp) and the lowest stimulator output intensity capable of inducing MEPs was recorded as threshold stimulation (TS). The time of onset to the stimulus response was measured as latency (Fig. 1D). We found no differences in latencies and TS between SCID and WT mice. However, the Amp of SCID mice was significantly higher than that of WT mice (Fig. 1D), suggesting better integrity of the corticospinal tract (CST), or increased excitability of the motor cortex, or even, better polysynaptic connectivity anywhere along the recording path of SCID mice after SCI.

After severe crush SCI, mice typically develop dense astrocytic/glial scars by two weeks that persist for at least eight weeks post-injury. The scar is thought to diminish the spread of inflammation and lesion volume (Anderson et al., 2016; Faulkner, 2004). However, glial scar at the epicenter of the injury is also a major impediment for regenerating axons to penetrate through to reconnect with distal spinal cord in order to resume neural control. We calculated the lesion size by measuring the volumn of epicenter between astroglial scar borders marked by GFAP immunostaining and found a significantly grearter lesion volumn in WT than SCID mice (Fig. 1E). Additionally, lectin blood vessel labeling also showed that SCID mice possessed more blood vessels with better morphology adjacent to the epicenter after SCI than WT mice (Fig. S1). Collectively, our findings provide clear evidence that SCID mice demonstrate less damage and better recovery after SCI.

### Spinal cord transcriptomic analysis revealed molecular differences between SCID and WT mice before and after SCI

**Figure 2:**
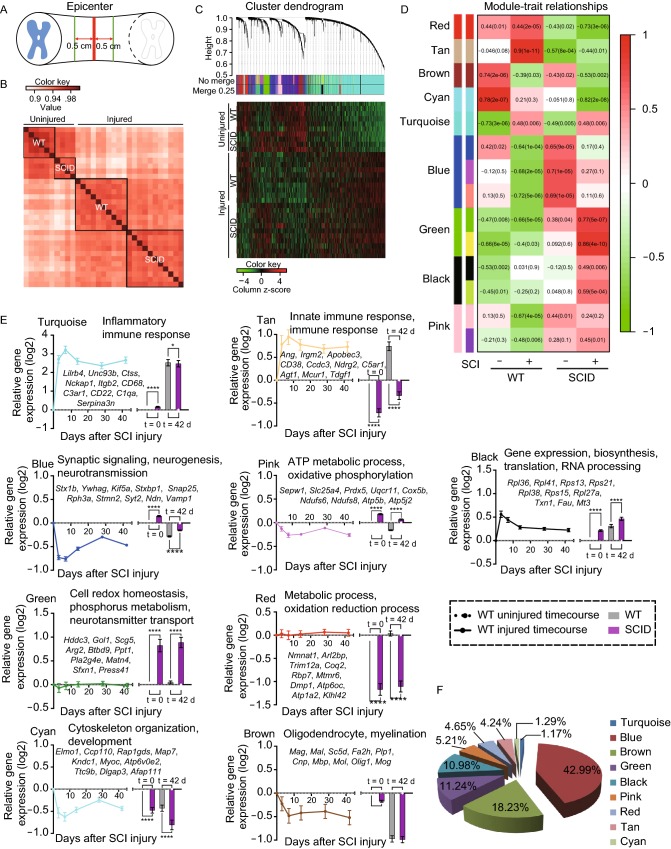
**WGCNA uncovered molecular mechanisms for better functional recovery in SCID mice**. (A) Total RNA was collected from 1 cm segments of spinal cord surrounding the epicenter of the lesion. (B) Unbiased hierarchical clustering heat map of the complete transcriptome dataset of 31 samples based on Pearson’s correlation coefficient. (C) Hierarchical cluster dendrogram of 31 samples showed coexpression modules identified using WGCNA. Modules corresponding to branches were labeled with colors indicated by color bands underneath the tree. 9 modules were detected after 0.25 threshold merge. (D) Module-trait correlation analysis revealed dynamic changes of nine modules under different conditions. (E) GO terms and gene expression time courses of 9 modules after SCI. The time course was described by average gene expression of top 30 hub genes within each module from injured WT mice from 0 to 42 days after SCI. Gene expression of 9 modules in uninjured and 42 d injured WT/SCID mice samples were displayed by bar graphs. Two sets of data (i.e., time course and WT/SCID comparisons) were normalized to uninjured WT group of each dataset, indicated by dash line at X-axis (Y = 0), for proper comparison between the two data sets. Hub genes of each module were also listed. (F) Total gene percentage pie-chart of each module. *P < 0.05, **P < 0.01, ***P < 0.001, ****P < 0.0001; statistical analyses were by Turkey’s multiple comparisons test (*n* = 31)

To explore potential molecular mechanisms by which immune deficiency promotes better functional recovery after SCI, we carried out extensive transcriptomic analysis. Adult SCID mice and their WT counterparts (20 g in body weight) were subjected to spinal cord crush injury at T9–10. 6 weeks post SCI, 10 WT mice and 11 SCID mice were sacrificed and 1 cm segments of spinal cord flanking the crush site (Fig. 2A) were collected for total RNA extraction. Uninjured spinal cord tissues were also collected from equivalent regions from 6 WT and 6 SCID mice, respectively. 2 SCID uninjured samples were excluded from further analysis due to poor sequencing data quality. Unbiased hierarchical clustering of the complete dataset based on Pearson’s correlation coefficient of whole transcriptomes among all samples divided samples into two major groups: uninjured and injured, suggesting SCI caused the most dramatic changes in gene expression. However, within both uninjured and injured groups, WT and SCID samples clustered separately, suggesting that immune deficiency also clearly affected gene expression with or without injury (Fig. 2B).

### WGCNA identified gene modules describing the effect of immune deficiency post SCI with biological validations

To understand which gene expression program changed as a result of SCI, and which change was due to immune deficiency, we used unbiased WGCNA on the complete dataset. 14 gene clusters/modules were identified, which were further merged into 9 modules according to module-module relationships (Fig. 2C). Module-trait relationships revealed that almost all 9 modules had different SCI-induced expression patterns depending on the genotype (Fig. 2D). To better evaluate expression changes of each module at different time points post SCI, 21 additional WT SCI samples were collected from 1, 3, 7, 14, 28 and 42 d post-injury for RNAseq. Averaged expression of the top 30 hub-genes from the above 9 modules was used to plot the time course of these modules post-injury (Fig. 2E). Bar plots were added to demonstrate module dynamic changes in WT and SCID samples before and 6 weeks after SCI. To make proper comparisons, two sets of data (i.e., time course and WT/SCID comparisons) were normalized to uninjured and WT samples within each group respectively.

In our previous work, we successfully used WGCNA on transcriptome data to describe pathological/biological events after complete spinal cord transection in rats (Duan et al., 2015). Intriguingly, our currently mouse study recapitulated the rat study by revealing that several modules with similar gene ontology (GO) terms follow similar temporal changes post-injury (Duan et al., 2015). For example, dramatically up-regulated immune responsive modules (turquoise and tan), up-regulated gene expression/biosynthesis module (black), as well as dramatically down-regulated synaptic signaling and neurotransmission module (blue), all appeared to represent major pathological events post injury regardless of injury type or animal species, indicating the robustness of transcriptomic analysis to digitally and objectively capture major pathological/ biological events after SCI.

Specifically, the turquoise module was rather big, containing about 43% of genes detected in this study (Fig. 2E and 2F). Expression of these genes quickly rose after SCI and stayed high 6 weeks after SCI. GO term and hub gene network analysis of this module indicated a linkage of this module to immune responses and cytokine production (Fig. S2B). Within the turquoise module, we found that the pan-leukocyte marker gene *Ptprc* (CD45) demonstrated statistically significant reduction in SCID as compared to WT after SCI. Additional key immune regulatory genes such as *Cd86* (a T cell activation antigen) and *Elf4* (a transcriptional activator involved in natural killer cell development and function) also demonstrated similar expression changes as *Ptprc*, consistent with the fact that SCID mice had reduced immune responses (Fig. S2D). Some of the classic T and B cell markers, such as CD3, CD4, CD8, CD79, had very low levels of expression in WT spinal cord samples, but still showed reduced expression in SCID especially after SCI (Fig. S2E). It is worth noting that immune function-related genes could be expressed in cells other than T or B cells, including microglia, or even neurons and astrocytes. Moreover, because of the cellular heterogeneity of spinal cord tissue, pooled transcriptomic analyses would not precisely reveal cell type-specific gene expression. The tan module, which is up-regulated with a very similar time course as the turquoise module post-injury, is also linked to immune response (*Ang2*, *Irgm2*, *Apobec3*, *Cd38*), innate immune response (*Ang2*, *Irgm2*, *Tdgf1*), and cell adhesion (*Tdgf1*, *Dock9*, *Stx3*). Unlike the turquoise module, the tan module showed dramatic decreases in SCID before and after SCI (Fig. S2F and S2G). Collectively, both turquoise and tan modules revealed attenuated immune responses in SCID compared to WT mice after SCI, which was predicted by the SCID genetic background (Fig. S2).

**Figure 3:**
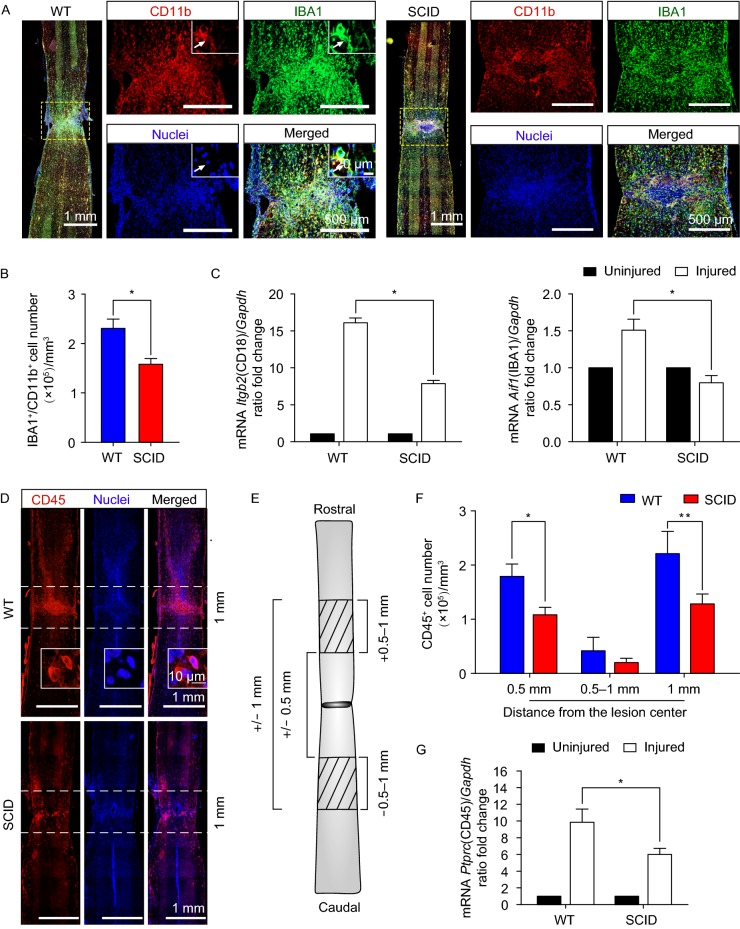
**Dampened immune response in SCID mice after SCI**. (A) Detection of microglia/macrophages in WT and SCID mouse spinal cord samples by IBA1 and CD11b immunostaining at 28 d post-injury. (B) Quantification of IBA1/CD11b double positive cells located within 1 mm of the spinal cord segment flanking the epicenter. (C) qRT-PCR validation of expression of inflammation-related genes *Itgb2*(CD18) and *Aif1*(IBA1) in WT and SCID mouse samples. (D) Immunostaining of pan-leukocyte surface marker CD45 in WT and SCID mouse samples at 28 d post-SCI. (F) Unbiased stereological quantification of CD45 positive inflammatory leukocytes in the three regions illustrated in (E) (mean ± SEM; *n* = 4, *P < 0.05, **P < 0.01, unpaired Student’s *t*-test). (G) qRT-PCR validation of *Ptprc*(CD45) gene expression in WT and SCID mouse samples

As expected, fewer CD11b and IBA1 double positive cells were found in the lesion epicenter in SCID mice than in WT mice (Fig. 3A and 3B), indicative of dampened immunue responses. We also evaluated a tan module hub gene *CD38*, which was a critical regulator of inflammation and innate immune responses (Partida-Sánchez et al., 2001). *CD38* had reduced expression in SCID mice after injury as measured by qRT-PCR (Fig. S2I), similar to *Aif1*(IBA1) and *Itgb2(*CD18) (Fig. 3C). Moreover, we used an anti-CD45 antibody to label both residential and infiltrating leukocytes to assess inflammation levels. Consistent with the RNAseq result, we found lower numbers of CD45^+^ leukocytes in SCID mice (Fig. 3F); qRT-PCR also confirmed the RNAseq result (Figs. 3G and S2D). Taken together, both transcriptomic data and immunohistochemistry evidence demonstrated that inflammatory immune responses were elevated significantly post-injury, and SCID mice displayed dampened adaptive and innate immune responses compared to WT mice.

The third module that was upregulated post-injury was the black module, suggesting enhanced gene expression, translation, and biosynthesis after SCI. This module peaked at day 3 post-injury. Compared to WT, SCID mice had heightened expression of genes involved in biosynthesis both before and after SCI, implying more active tissue reconstruction in SCID mice (Fig. S3A–C). In contrast, energy and ATP metabolic process-related pink module genes decreased expression post-injury. However, compared to WT mice, SCID mice had increased expression of genes in the pink module both before and after SCI. In summary, upregulation of biosynthesis and energy metabolism-related genes in SCID mice indicated more active bio-construction, which might help functional recovery after SCI (Fig. S3D–G).

There is a module dedicated to oligodendrocyte and myelination genes (the brown module), which was downregulated after SCI, consistent with de-myelination (Fig. S4A–C). However, quantitative RT-PCR of *Plp1* mRNA showed higher expression in SCID samples post SCI (Fig. S4D and S4E), thus it is possible that remyelination is enhanced in SCID. The cyan module is the smallest module with a weakly significant GO term related to cytoskeleton organization. This module is decreased after SCI (Fig. S4F–H).

**Figure 4:**
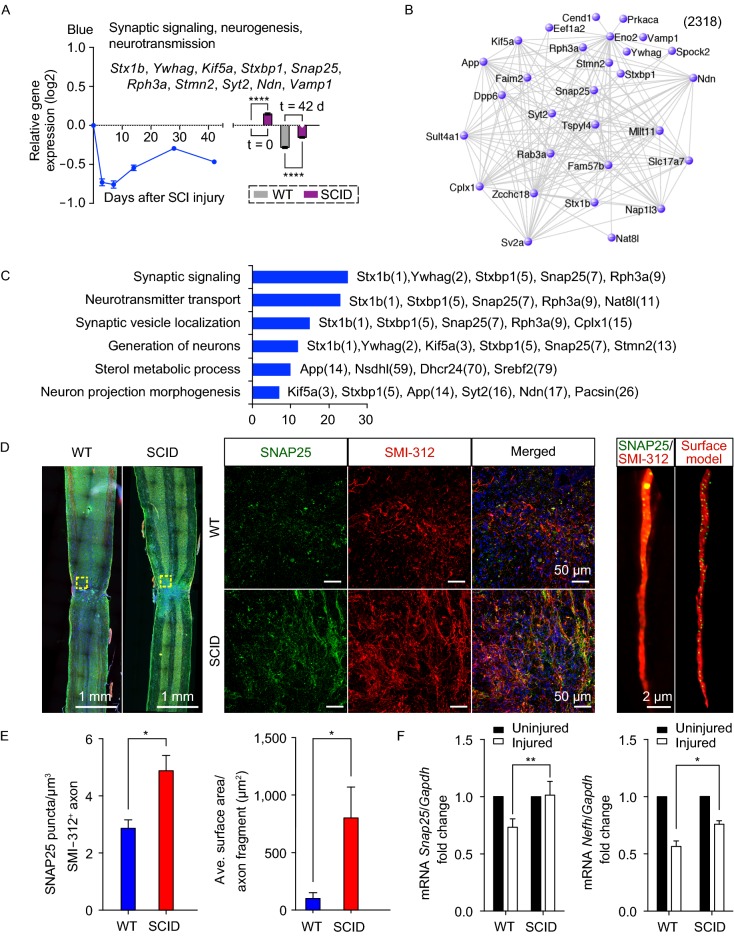

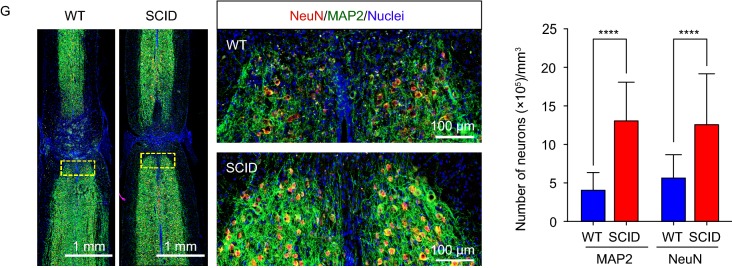
**Dampened Immune response in SCID mice after SCI was associated with enhanced neural protection and neurotransmitter release**. (A) Time course of blue module gene expression in WT mice post SCI, as well as overall module expression in uninjured and 42 d injured WT/SCID samples (bar graphs). (B) Blue module hub gene network constructed by top 30 hub genes. (C) GO Terms of the blue module with representative genes associated with each term. Numbers in parentheses indicated rankings within the module calculated by their correlations with the eigengene (representative module gene) of this module. (D) The axons adjacent to the epicenter were labeled by a pan-axon filament marker, SMI-312. The presynapses were lableded by a presynaptic marker, SNAP25. The middle panels showed high magnification of highlighted areas in the left panels. Right panels showed representarive images of SNAP25 and SMI-312 immunostaining and the surface model that were used for presynaptic puncta quantification. The surface model was generated by Imaris software (Bitplane). (E) The quantification of SNAP25^+^ puncta within SMI-312^+^ labeled axons (per µm^3^) adjacent to the injury epicenter (left panel) (mean ± SEM; *n* ≥ 15, *P* < 0.05, Mann Whitney test). The average surface area (μm^2^) per SMI-312 positive axon fragment was calculated to illustrate the integrity of axon adjacent to the epicenter (right panel). (F) qRT-PCR validations of *Snap25* and *Nefh* genes expression in 1cm spinal cord segments of WT and SCID mice. (G) Immunostaining of neuronal makers MAP2 and NeuN in WT and SCID mouse spinal cord samples at 28 d post-injury, with quantifications. High magnification images of highlighted regions are presented on the right. (mean ± SD; *n* ≥ 15, ****P < 0.0001, Mann Whitney test)

The blue module is the second largest module in the data set, and contains genes involved in synaptic signaling, neurogenesis, and neurotransmission. In WT mice, this module underwent an immediate decrease after SCI and recovered somewhat after 2 weeks. Compared to WT mice, this group of genes were expressed at significantly higher levels in SCID mice post-injury (Fig. 4A–C). To assess the differences between the two groups, we carried out immunostaining and qRT-PCR of *Snap25* and *Nefh* (neurofilament heavy polypeptide). As expected, SCID mice possessed a greater number of SNAP25^+^ puncta and less fragmented neurofilament processes adjacent to the epicenter compared to WT mice after SCI (Fig. 4D–F). Moreover, immunostaining of the pan-neuronal markers Map2 and NeuN (*Rbfox3*) revealed more NeuN^+^/Map2^+^ neuronal entities adjacent to the lesion epicenter of SCID mice with less fragmented neuronal processes (Fig. 4G). Taken together, this evidence suggests that immune deficiency in SCID mice leads to better neural protection as demonstrated by less injury-induced axonal fragmentations, which could potentially explain better motor functional recovery in SCID mice after SCI.

### WGCNA established gene modules revealing the impact of immune deficiency on normal CNS function without injury

Interestingly, all 9 modules demonstrated significant differences between WT and SCID samples before injury, indicating that immune deficiency would likely affect CNS functional state under normal physiological conditions without injury. Red and green modules, which were not affected by injury, displayed the most significant differences between SCID and WT mice (Figs. 2, S5A and S5B). The green module is linked to cell redox homeostasis and neurotransmitter transport (Fig. S5B and S5C). Its heightened expression in SCID is consistent with a stronger program in neurotransmission. In contrast, the red module reduced expression in SCID mice. It is involved in various metabolic processes and the oxidation reduction process (Fig. S5E–G). *Dmp1* is one of the hub gene in the red module, which is recently found to be widely expressed in CNS neurons and glia, and one of its functions is linked to the blood-brain-barrier (Jing et al., 2017).

**Figure 5:**
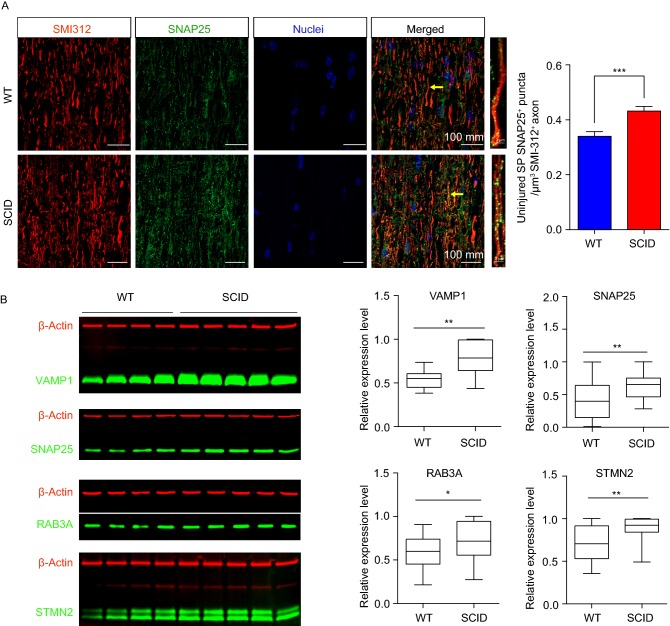
**Uninjured SCID mice displayed boosted synaptic transmission program**. (A) Immunostaining of SNAP25 and SMI-312 in WT/SCID uninjured spinal cord samples with high magnification images of indicated areas presented to the right. SNAP25^+^ puncta within SMI-312^+^ labeled axons (per µm^3^) of uninjured samples in the equivalent location as injured samples were measured (mean ± SEM; *n* ≥ 20, ***P < 0.001, Mann Whitney test). (B) Western blotting analysis of blue module hub genes VAMP1, SNAP25, RAB3A, STMN2 and β-actin protein levels of uninjured spinal cord sample from WT and SCID mice (mean ± SD; *n* = 4 (WT), *n* = 5 (SCID), *P < 0.05, **P < 0.01, unpaired Student’s *t*-test)

**Figure 6:**
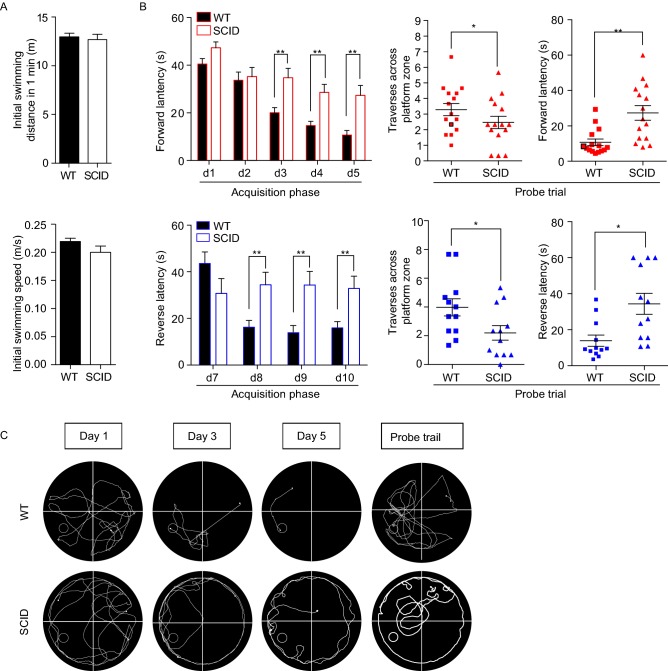
**Uninjured SCID mice showed worse behavior performance in MWM**. (A) The test of initial swimming distance (m) and swimming speed (m/s) before training showed no significant differences between two genotypes (mean ± SEM; *n* = 15, *P* > 0.05, unpaired Student’s *t*-test). (B) Upper panels (in red) shows the escape latency for mice to reach the hidden platform during forward acquisition training phases, as well as traverses through the platform zone and the latency to first reach the platform zone (mean ± SEM; *n* = 15, *P < 0.05, **P < 0.001, unpaired Student’s *t*-test). The lower panel (in blue) shows the exact same results in reverse test (acquisition phase and probe trial). (C) Representative swimming traces of SCID and WT mice during acquisition training phases and probe trial

Most interestingly, we observed increased expression of blue module genes even before injury, suggesting that immune deficiency could disturb/affect neurotransmission program without injury (Fig. 4A). Quantification of immune-stained SNAP25^+^ puncta within SMI312 (*Nefh*)^+^ neurofilament in uninjured samples further suggested enhanced synaptic transmission capacity in SCID mice under normal conditions (Fig. 5A). Moreover, immunoblotting of blue module hub genes VAMP1, SNAP25, RAB3A, STMN2 (Fig. 5B) also confirmed upregulation of synaptic transmission related genes in SCID mice under uninjured conditions. An imbalance of neurotransmission or hyper-connectivity has been postulated to contribute to many brain disorders including schizophrenia and autism (Supekar et al., 2013; Han et al., 2014; Hook et al., 2014; Whitfield-Gabrieli et al., 2009). The abnormal neural transmission program let us predict that uninjured SCID mice might display behavioral deficits. To test this hypothesis, we subjected uninjured SCID and WT mice to the Morris water maze (MWM), which is a spatial learning and memory task requiring mice to locate a hidden platform in an opaque pool using visual cues in the testing room. To determine potential differences in motor ability between the two genotypes, we tested the initial swimming distance and swimming speed before training trials; no significant difference between two groups was detected (Fig. 6A). Acquisition of spatial learning and memory in WT mice was observed as daily reduced latency in finding the platform. Statistically significant differences in latency were observed between two groups at day 3 of training and onward towards the end of acquisition phase at day 5 (Fig. 6B, red bars in upper left panel), which indicated significantly attenuated learning ability in SCID mice. To assess reference memory, a probe trial was performed with a novel start position implemented and the platform removed at day 6, which was also 24 h after acquisition phase. SCID mice showed diminished memory recall, as indicated by fewer transverses across the platform zone, longer latency to first enter the platform zone (Fig. 6B, two upper right panels in red). Likewise, compared to WT mice, SCID mice performed more poorly in reverse trials (Fig. 6B, lower panels in blue). Moreover, WT mice learned to swim away from the pool wall to search for the platform in the inner part, whereas SCID mice swam aimlessly in circles along the pool wall during both the acquisition phase and the probe trial (Fig. 6C), which indicated impairment in learning and memory of SCID mice (Kipnis et al., 2004). Interestingly, it has been reported that mice with immunodeficiency have inferior neurogenesis and reduced spatial learning abilities (Ziv et al., 2006). They also have cognitive and social impairment (Kipnis et al., 2004; Filiano et al., 2016), which may be caused by hyper-connectivity in the prefrontal cortex (PFC) (Filiano et al., 2016, 2017). Collectively, all these observations suggest that neurotransmitter imbalance or disturbed synaptic connectivity in SCID mice would affect normal CNS function under physiological conditions.

## Discussion

SCI in humans is a tremendously challenging medical condition, currently with no cure. The SCI research field harbors substantial controversy because there are many SCI models using different species with varying surgical manipulations. Even within mouse models, the genetic background of the mouse strains used in the studies could interfere with experimental observations (Luchetti et al., 2010; Kigerl et al., 2006). Moreover, some of the classic behavioral assays such as BBB or BMS scoring rely on human judgment, contributing to irreproducibility of some of the studies. The field needs more objective and quantitative assessment. Big data-based analyses, which take into account a large repertoire of biological elements including genetic, epigenetic, and environmental factors, could be implemented to objectively and systematically reflect various aspects of pathological events. Transcriptomic analysis is one such approach, which has begun to manifest its sensitivity, objectivity and power to discover in the study of injury and disease. More importantly, as transcriptome data are deposited into the public data bases upon publication of the study, which literally display the whole SCI study for public scrutinization, because anyone, at anytime and anywhere, can independently mine the transcriptome data to reveal the nature and various perspectives of how the study was performed. This approach will also benefit comparative studies from labs to labs, time to time, and studies to studies in an objective way, which may resolve the inconsistency and irreproducibility issues in the SCI research fields.

In the current study, using unbiased transcriptomic analysis of uninjured and post-injury spinal cord tissues from WT and SCID mice via WGCNA, we found that transcriptome changes were capable of revealing molecular differences caused by immune deficiency and by SCI. By partitioning the whole transcriptome dataset into 9 different gene clusters (modules), immune deficiency predicated by the SCID genotype could be easily identified. Moreover, dampened adaptive and innate immune responses after SCI in SCID mice were also obvious. More importantly, this approach is sensitive enough to capture the dynamic changes of neurotransmission, synaptic signaling, and neurogenesis-related gene programs (blue module) after SCI in mice with differing genetic backgrounds. The energy metabolism-related pink module also had a similar pattern (Fig. S3D–G). Given that neurons have high demand for mitochondrial function or energy metabolism, elevated pink module gene expression may contribute to heightened function in neurotransmission and hyper-connectivity. Another neurotransmitter transportation-related module is the green module. Interestingly blue, pink and green modules all demonstrated elevation in SCID samples without SCI, indicating that immune function and neuronal function have an intimate relationship even without injury, which might explain why SCID mice performed poorly in MWM test and why SCID mice are hypo-responsive to social stimuli (Filiano et al., 2016).

During our experiments, we observed individual variations in animal behavior (e.g., BMS scoring) even within the same genotype. Given we carried out transcriptomic analyses on animals that had completed the behavioral analyses, transcriptome information from each mouse could be paired with the corresponding behavioral data. We then identified genes that are purely positively or negatively correlated with BMS scores in WT and SCID samples ignoring genotype differences. To our satisfaction, immune responsive and inflammatory responsive genes are most negatively correlated with good BMS scores (Fig. S6). On the contrary, neurogenesis and neurotransmission-related genes are most positively correlated with better behavior. This additional analysis is in agreement with the salient finding from WGCNA.

**Figure 7:**
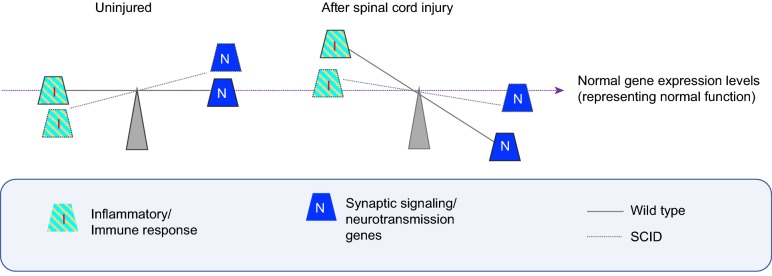
**Current working model delineating the seesaw relationship between neurotransmission program and immune function**. As demonstrated, synapse/neurotransmission-related gene expression program can be influenced by immune functional gene expression, and an appropriate (i.e., neither too high nor too low) expression levels of synapse/neurotransmission module genes are critical for proper neuronal function

Even though anti-inflammation or immunosuppressive drugs, such as glucocorticoids and methylprednisolone (MP), have been widely considered as appropriate SCI treatment since the last century, whether they are actually beneficial has not been systematically investigated. In this study, we identified major molecular programs that changed after SCI based on unbiased transcriptomic analyses, which strongly suggested that dampened inflammation in SCID mice would lead to better functional recovery. This objectively demonstrated the beneficial role of suppressing inflammation after CNS injury. As depicted in our seesaw model (Fig. 7), we revealed that the neural transmission program could be strongly modulated by immune functions. In addition, appropriate (neither too high nor too low) expression levels of synapse/neurotransmission module genes are critical for proper neuronal function. Since changes in immune responses are also temporally specific, it would be ideal to profile SCID mouse spinal cord at different time points after injury, through which we might find the best time window for immune modulation. Moreover, recently developed massive single cell transcriptome profiling could be unutilized in the near future to handle heterogenous cellular composition in whole spinal cord samples, and precisely reveal cell-type specific molecular changes after SCI. Finally, whether MP had additional effect other than immune suppression, e.g., inhibition of neurogenesis, could also be objectively and systematically investigated by transcriptomic analysis, especially by means of single cell transcriptomic analysis.

Traditionally, SCID mice were often used for studies on cell or stem cell transplantations to promote neural repair. From this work, we clearly demonstrated that the functional state of brain and spinal cord in SCID mice is significantly different from that of WT mice. Therefore, additional caution should be taken when using SCID mice, because results may not be translatable to WT conditions. Moreover, several clinical investigations reported that disturbed neural circuit homeostasis, resulting in hyper-connectivity, exists in several neurodevelopment disorders (NDDs) including autism spectrum disorder, schizophrenia, and Rett syndrome. These NDDs are also accompanied with immune abnormalities, imbalance in neurotransmission, and cognitive impairment (Supekar et al., 2013; Fornito et al., 2012; Nott et al., 2016; Liu et al., 2017) (Fig. 7). All of these provide a rationale for potentially treating NDDs via immune modulation. Overall, our findings provide a new starting point to understand the bidirectional interactions between the neural and immune systems, and potentially providing a base for treating neurological disorders via immune modulations.

## Materials and methods

### Animal surgery

All experimental procedures were approved by and performed in accordance with the standards of the Animal Welfare Committees of Tongji University in Shanghai China and UCLA. 8–10 week-old mice of both male and female were used with body weights between 20–24 g. Anesthesia was induced in WT mice (C57BL/6) and SCID mice with intraperitoneal injection of sodium pentobarbital (10 mg/mL, 70 mg/kg) and surgery was performed using a dissecting microscope (Nikon). A laminectomy was performed at T9, and followed by spinal cord crush using a modified bulldog artery clamp with 60 g constant front-end force persisting for 3 s. For the uninjured groups, only laminectomy of T9 vertebra was performed without the subsequent crush injury. Bladders of mice were expressed manually twice per day for the first week post-injury, and then once a day thereafter. Animals were daily monitored to avoid infection, abnormal wound healing or body weight drop.

### Behavioral analysis

#### Open field test

Gross voluntary use of the hindlimbs of WT and SCID mice were evaluated by the Basso Mouse Scale (BMS) open field test. Mice were evaluated before injury and at 1, 7, 14, 21, 28, 35, and 42 d post injury by the same two observers without awareness of experimental conditions in a double-blinded manner.

#### Rotarod test

Balance and motor function of WT and SCID mice were measured using a rotarod. Mice were trained in 2 trials a day for 7 continuous days before testing. First, mice were placed on the rod at the initial speed of 0 rpm, and then accelerated gradually to 40 rpm. The speed (in rpm) when mice fall from the rod was recorded as the drop-off speed. Second, mice were placed on the rod at a constant speed of 15 rpm. The time (in seconds) at which the mice fell off was recorded as the drop-off time.

#### Morris water maze test

Water-maze testing was performed as described previously (Vorhees and Williams, 2006; Petitto et al., 1999). One hour before the test, WT and SCID uninjured mice (8 weeks) were taken to the 21–23 °C MWM testing room, which was equipped with visual cues on the walls. The water maze consists of a circular pool (diameter: 100 cm, height: 45 cm). It was filled with opaque water and an escape platform was placed in one of four quadrants. During the 5-day acquisition phase, all mice were given 4 trials per day, when they were released to the pool from four different starting positions. The latency to reach the platform and the swim path length were recorded by tracking video and were analyzed by ANYmaze software. The interval between trials was 10 min. To assess reference memory, a probe trial was performed at day 6 (24 h after the last acquisition trial), where a random start position was implemented and the platform was removed. Three assessments were performed by recording the latency for mice to first enter the platform zone, the number of traverses made across the platform zone, and the total time spent in the platform zone. In addition, we carried out reverse testing in the next 5 days to confirm the initial test results. All procedures were exactly the same for reverse test as for the forward test, except that the platform was relocated in the opposite quadrant of the pool.

### Electrophysiology analysis

Electrophysiological testing was performed for each group at 42 d post-injury using keypoint-II bichannel evoked potential/electromyography (Dantech). Mice were recorded before injury as uninjured controls (*n* = 5). All the animals were anesthetized by intramuscular (IM) injections of ketamine (20 mg/kg). For MEP recording, two stimulating electrodes were included: the positive electrode was placed on the skull surface of motor area of cerebral cortex (AP + 1.0, L/R ± 1.5, DV 0, mm from Bregma), 1 mm behind the bregma and 1.5 mm on the left or right side from the midline; and the negative electrode was placed on the skull 0.5 cm lateral to the positive electrode. The recording electrode was inserted into the left or right gastrocnemius muscle of hind limbs with a depth of 1.5 mm. Moreover, the reference electrode was placed at 2 cm away from the recording electrode and the grounding line was placed in the middle of the stimulating electrode and recording electrode. 0–10 mA single square wave (1 Hz) was applied to stimulate the motor area of the cerebral cortex through skull with a duration of 0.2 ms. Three features of MEP were recorded at the gastrocnemius muscle of the hindlimb, i.e., peak-to-peak amplitudes were calculated as amplitude values of MEP (Amp) and the lowest stimulator output intensity capable of inducing MEPs were recorded as threshold stimulation (TS). The onset time of the first response to the stimulus was measured as latency (Nardone et al., 2015).

### RNA-Seq

Total RNA was extracted from a 1 cm spinal cord segment flanking the crush injury site from wild type and SCID mice at different time points after injury using TRIzol (Invitrogen) and then purified by RNeasy Mini Kit (QIAGEN) according to the manufacturer’s instructions. 1–3 µg qualified RNA was subjected to Illumina TruSeq RNA Library Prep Kit v2 for library preparation, followed by Hiseq 2000 SE50bp Sequencing. The quality of RNA and library were assessed by Agilent Bioanalyzer 2100.

### WGCNA

A signed weighted correlation network was constructed using 31 samples by first creating a matrix of pairwise correlations between all pairs of genes with annotation. The resulting Pearson correlation matrix was transformed into a matrix of connection strengths (e.g., an adjacency matrix) using a power of 12 (Langfelder and Horvath, 2008). Then the topological overlap was calculated to measure network interconnectedness (Yip and Horvath, 2007). For each dataset, we used average linkage hierarchical clustering to group genes on the basis of the topological overlap dissimilarity measure (1-topological overlap) of their network connection strengths. Using a dynamic tree-cutting algorithm and merging threshold function at 0.25, we identified 9 modules.

### Identification and visualization of hub genes

We summarized the gene expression profile of each module by module eigengene (i.e., the first principal component obtained by singular value decomposition). We then defined each gene’s membership corresponding to each module as the Pearson correlation between the expression level of the gene and each module’s eigengene, also known as module eigengene-based connectivity (kME). This measure was naturally scaled to lie in the interval [− 1, 1]. Genes with the greatest module membership values are referred to as intramodular hub genes. We used VisANT (version 1.0) to visualize the top 30 genes’ connections (based on topological overlap). Intramodular hub genes (i.e., genes with the highest kME values) usually are centrally located inside each module’s network (Duan et al., 2015). The top 30 hub-genes average expression of 9 modules was also extracted from 21 wild type mice samples collected from 7 different time points post SCI to plot temporal changes of these 9 module genes expression after SCI in WT spinal cord.

### GO analysis

Functional annotation was performed with the Database for Annotation, Visualization and Integrated Discovery (DAVID) bioinformatics resource. Genes with kME > 0.75 within each module were subjected to this analysis.

### qRT-PCR

1 cm spinal cord segment flanking injury sites at T9–10 were dissected from WT and SCID mice at 42 d post injury for RNA extraction. RNA from uninjured spinal cord tissues were also collected from WT and SCID mice at the same location. Total RNA was isolated with TRIzol and purified by RNeasy Mini Kit (Invitrogen) according to the manufacturers’ instructions. cDNA was generated using PrimeScript RT reagent kit with gDNA removal (TAKARA). Real-Time PCR was performed with TAKARA SYBR Premix Ex TAQ (Tli RNaseH Plus) Kit using ABI 7900 Sequence Detector (Applied Biosystems). PCR efficiency and specificity of each primer pair was examined by a standard curve of serially diluted cDNA and melting curve functionality respectively. Fold change was calculated based on 2^−ΔΔCt^ method after normalization to the transcript level of housekeeping gene *Gapdh*. Primer sequences used in the RT-PCR are as followed:

**Table:** Primers for qRT-PCR

Gene	Primer	Length	Direction
*Gapdh*	ACCCAGAAGACTGTGGATGG	20	Forward
	CACATTGGGGGTAGGAACAC	20	Reverse
*Ptprc*(CD45)	TGGAATGACCTCAAGGTGTCCTC	23	Forward
	GCTGTACACACCCACAGCACTCTTA	25	Reverse
*Aif1*(IBA1)	AGCTGCCTGTCTTAACCTGCATC	23	Forward
	TTCTGGGACCGTTCTCACACTTC	23	Reverse
*Itgb2*(CD18)	TGCATATGTGACGAAGGCTACCA	23	Forward
	ACTTCAGGCACTCGGCACAA	20	Reverse
*Itgax* (CD11c)	TATCGTGGGCAGCTCAGTGG	20	Forward
	GCGGGTTCAAAGACGATGG	19	Reverse
*Snap25*	CGGCATCATCGGAAACCTC	19	Forward
	GCACGTTGGTTGGCTTCATC	20	Reverse
*CNPase*	GTGCTGCACTGTACAACCAAATTC	24	Forward
	TCCTGATCGGTCAGCACCAC	20	Reverse
*Plp1*	AGATTCTGCCAGCTGTTAGCTGTTC	25	Forward
	AGGTTTCAAATTCTGCCTGTCCTC	24	Reverse
*Nefh*	GTTCCGAGTGAGGTTGGACC	20	Forward
	CCGCCGGTACTCAGTTATCTC	21	Reverse

### Immunohistochemistry

Animals were euthanized by overdose of pentobarbital. After transcardial perfusion with PBS and 4% paraformaldehyde, spinal cords were excised under a dissection microscope and post-fixed in paraformaldehyde at 4 °C for another 8 h, followed by cryoprotection using 30% sucrose. The spinal cord tissue including lesion area was embedded in OCT compound and sliced horizontally to produce 15 µm frozen sections using a cryostat microtome (Leica). Fluorescence immunohistochemistry was performed using following primary antibodies: rabbit anti-glia fibrillary acid protein (GFAP, 1:1,000, Dako), rat anti-CD45 (ebioscience, 1:1,000), rat anti-CD11b (ebioscience, 1:500), rabbit anti-IBA1 (WAKO, 1:500), rabbit anti-SNAP25 (Synaptic Systems GmbH, 1:1,000), purified anti-Neurofilament Marker (pan axonal, cocktail, SMI-312) (Biolegend, 1:1,000), mouse anti-CNPase (Abcam, 1:1,000), rabbit anti-PLP1 (Abcam, 1:1,000), fluorescence labeled Lectin (Vector, 1:20), chicken anti-MAP2 (Abcam, 1:1,000), rat anti-NeuN (Abcam, 1:1,000).

### Western blot analysis

1 cm uninjured spinal cord sample were dissected and collected from WT and SCID mice as described in qRT-PCR segment. Such tissue samples were homogenized in 500 μL RIPA buffer (Beyptime, P0013K) with 1 mmol/L phenylmethanesulfonyl fluoride (PMSF) and a mixture of protease inhibitor cocktail. After 10 min incubation in ice, they were then centrifuged at 12,000 rpm for 30 min at 4 °C and quantitated using a BCA quantification kit. 25 μg protein of each sample was loaded on 10% acrylamide gels. The antibodies used for western blotting were rabbit anti-VAMP1 antibody (Abcam), rabbit anti-SNAP25 (SYSY), rabbit anti-RAB3A antibody (Abcam), rabbit anti-STMN2 antibody (Abcam), mouse anti- β-Actin antibody (CST), 800CW goat-anti-rabbit IgG (LI-COR), 680LT goat-anti-mouse IgG (LI-COR). Western blots were imaged using Odyssey Imaging System (LI-COR Biosciences).

### Image quantification

Cell counts, lesion span, co-localization analysis, spot counting and axon surface modeling were determined using unbiased sampling and Imaris software (Bitplane). All figures were composed with adobe Photoshop, adobe Illustrator and Coreldraw. To study the level of inflammation post injury, CD45/IBA1/CD11b immunostaining images were acquired by 20× objective lens without optical zoom (Plan-Apochromat, NA = 0.8) by Zeiss LSM800 at a resolution of 1,024 × 1,024 pixels. A z-step of 1.5–3 μm interval was used. CD45^+^ leukocyte cells in 1 mm or 2 mm spinal cord segment flanking the epicenter were counted. The number of immune activated microglial cells were determined by counting IBA1/CD11b double positive cells within 1 mm spinal cord segment flanking the epicenter. To quantify SNAP25 expression in the WT and SCID mice after SCI, 63× oil immersion objective lens was applied (Planapo, Zeiss; NA = 1.4) to take z-step images at a resolution of 1,024 × 1,024 pixels. A z-step of 0.25–0.3 μm interval was used. The puncta were analyzed as described by Fogarty (Fogarty et al., 2013). Briefly, the SMI-312 positive axons were marked by the Imaris create surface tool. The SNAP25^+^ puncta with diameter of ~ 0.25 μm located along the SMI-312 labeled axon surface were selected. The number of SNAP25^+^ puncta per μm^3^ of axon fragment was calculated by the Imaris spot analysis tool (SCID *n* = 15, WT *n* = 18, Mann Whitney test, *P < 0.05). The average surface area (μm^2^) per SMI-312 positive axon fragment was calculated to illustrate the integrity of axon adjacent to the epicenter (SCID *n* = 13, WT *n *= 8, Mann Whitney test, ***P < 0.001). For MAP2 and NeuN quantification, we acquired the images using 20× objective lens without optical zoom (Plan-Apochromat, NA = 0.8) by Zeiss LSM880 at a resolution of 1,024 × 1,024 pixels. The cells were scanned at 1.5–2.5 µm intervals along the z-axis. A final high-definition 3-D image of dendrite was achieved via reconstructing these consecutive scans using software Imaris (Bitplane). From which, the number of the MAP2^+^ and NeuN^+^ neurons in the area 200–850 µm away from the epicenter (excluding the epicenter) were quantified. Vessel-like Map2 signals that do not co-localize with DAPI labeled nucleus are not counted.

### Statistical analysis

Statistics were carried out using Graphpad Prism software. The Student’s *t*-test of the Mann-Whitney *U* test and unpaired Student’s *t*-tests were used to determine statistical differences between two groups. *P* < 0.05 was considered to indicated a statistically significant difference. Data were presented as mean ± SEM.

## Electronic supplementary material

Below is the link to the electronic supplementary material.

Supplementary material 1 (PDF 1623 kb)
