# Supplementary material for: Unbiased transcriptomic analyses reveal distinct effects of immune deficiency in CNS function with and without injury
Source: Protein Cell. 2018 Jun 28;10(8):566–82. doi: 10.1007/s13238-018-0559-y (PMC6626597; doi:10.1007/s13238-018-0559-y)
Supplement: Supplementary file 1 — Supplementary material 1 (PDF 1623 kb) [file 13238_2018_559_MOESM1_ESM.pdf]

Sup. Fig1. SCID mice showed better preservation of blood vessels after SCI.
(A) Labeling of blood vessels by lectin in WT andSCID mouse spinal cord samples at 28d post-injury. Magnified images of highlighted regions are shown on the right.

Sup. Fig2. GO terms and hub genes networks of turquoise and tan modules.
Time course of turquoise **(A)** and tan **(F)** module gene expression in WT mice post SCI, as well as their expression in uninjured and 42d injured WT/SCID samples (bar graphs). Top six GO Terms of turquoise and tan modules were shown in **(B)** and **(G)**, respectively, with representative genes associated with each term. Numbers in parentheses indicated rankings of genes within the module calculated by their correlations with the eigengene (representative module gene) of each module. Hub gene networks of the turquoise **(C)** and tan **(H)** modules were constructed by top 30 hub genes in each module. **(D)** Expression levels of Immune function-related genes in the turquoise module demonstrated reduction in SCID samples after SCI. **(E)** Typical T and B cell markers with very low expression levels in WT spinal cord samples still showed a trend toward reduction in SCID samples before and after SCI. (Mean ± SEM *p<0.05, ****p<0.0001, n=31 except in I, n=6) **(G)** qRT-PCR validation of a tan module hub gene *CD38* in WT and SCID mouse spinal cord samples.

Sup. Fig3. GO terms and hub gene networks of black and pink modules.
Time course of black **(A)** and pink **(D)** module gene expression in WT mice post SCI, as well as their expression in uninjured and 42d injured WT/SCID samples (bar graphs). Top six GO Terms of black and pink modules were shown in **(B)** and **(E),** respectively, with representative genes associated with each term. Numbers in parentheses indicated rankings of genes within the module calculated by their correlations with the eigengene (representative module gene) of each module. Hub gene networks of the black **(C)** and pink **(F)** modules were constructed by top 30 hub genes in each module. **(G)** qRT-PCR validation of pink module hub gene *Ndusf4* and *Atp5b* expression in WT and SCID mouse samples.

Sup. Fig4. GO terms and hub gene networks of brown and cyan modules.
Time course of brown **(A)** and cyan **(F)** module gene expression in WT mice post SCI, as well as their expression in uninjured and 42d injured WT/SCID samples (bar graphs). Top six GO Terms of brown and cyan modules were shown in **(B)** and **(G),** respectively, with representative genes associated with each term. Numbers in parentheses indicated rankings of genes within the module calculated by their correlations with the eigengene (representative module gene) of each module. Hub gene networks of the brown **(C)** and cyan **(H)** modules were constructed by top 30 hub genes in each module. **(D)** Immunostaining of oligodendrocyte markers PLP1 and CNPase on WT and SCID mouse spinal cord samples at 28d post-injury. **(E)** qRT-PCR validation of brown module hub gene *CNPase* and *Plp1* expression in WT and SCID mouse spinal cord samples.

Sup. Fig5. GO terms and hub gene networks of green and red modules.

Time course of green **(A)** and red **(D)** module gene expression in WT mice post SCI, as well as their expression in uninjured and 42d injured WT/SCID samples (bar graphs). Top six GO Terms of green and red modules were shown in **(B)** and **(E),** respectively, with representative genes associated with each term. Numbers in parentheses indicated rankings of genes within the module calculated by their correlations with the eigengene (representative module gene) of each module. Hub gene networks of the green **(C)** and red **(F)** modules were constructed by top 30 hub genes in each module. **(G)** qRT-PCR validation of red module hub gene *Dmp1* expression in WT and SCID mouse spinal cord samples.

Sup. Fig6. Paired correlation analysis of mouse gene expression with behavioral outcomes (BMS) across 46 mouse samples from different experiments and profiled at different times. Data indicated that mice with better functional recovery have lower expression of immune function-related genes and higher expression of neurogenesis & neuro-transmission-related genes. (A) GO terms of top 500 genes negatively and positively correlated with BMS scores. (B) Heat maps of representative genes, whose expressions that are negatively (left panel) and positively (right panel) correlated with BMS scores. Scale bars for BMS scoring and gene expression levels were presented.

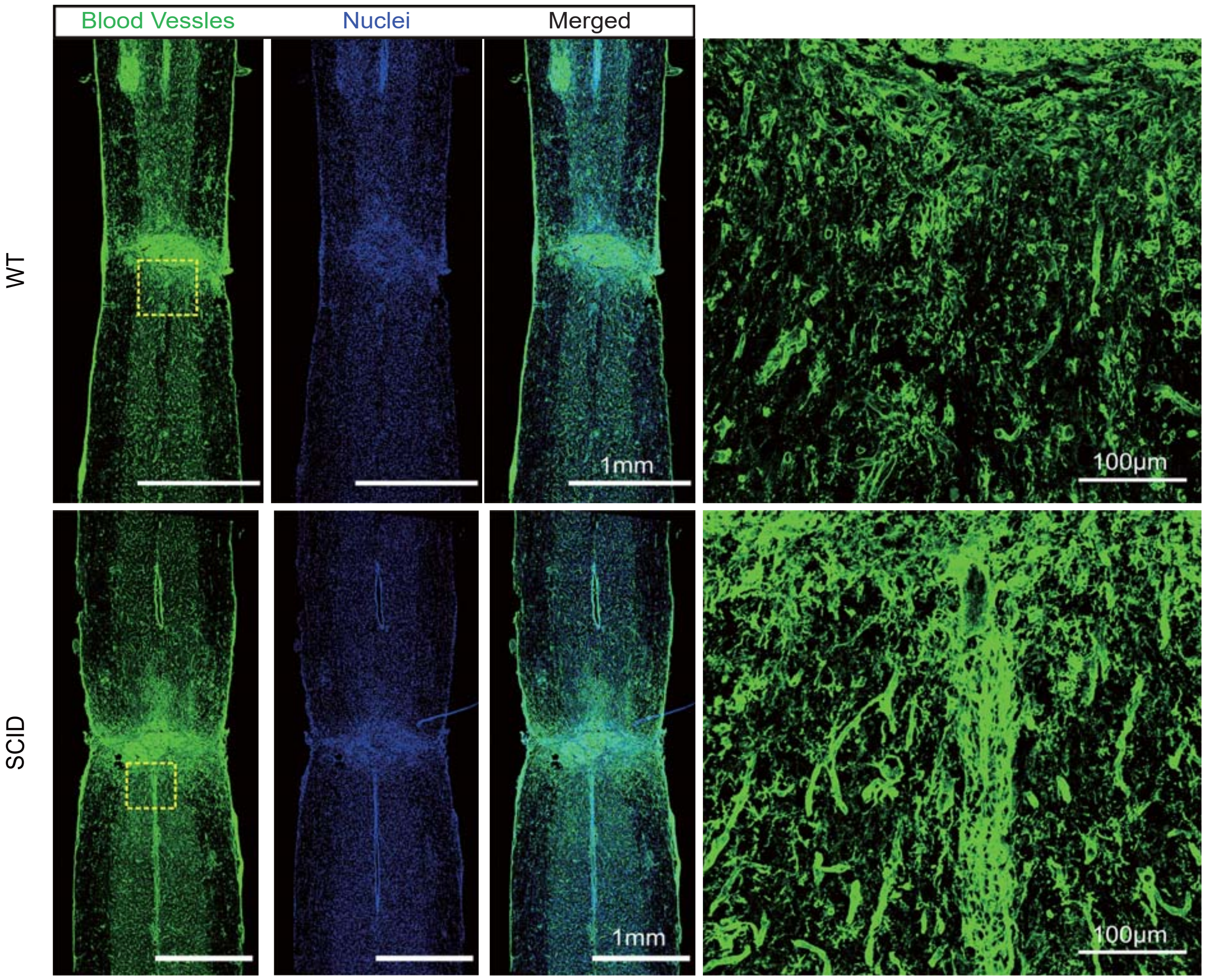

Sup. Fig 1 Luo *et al*.

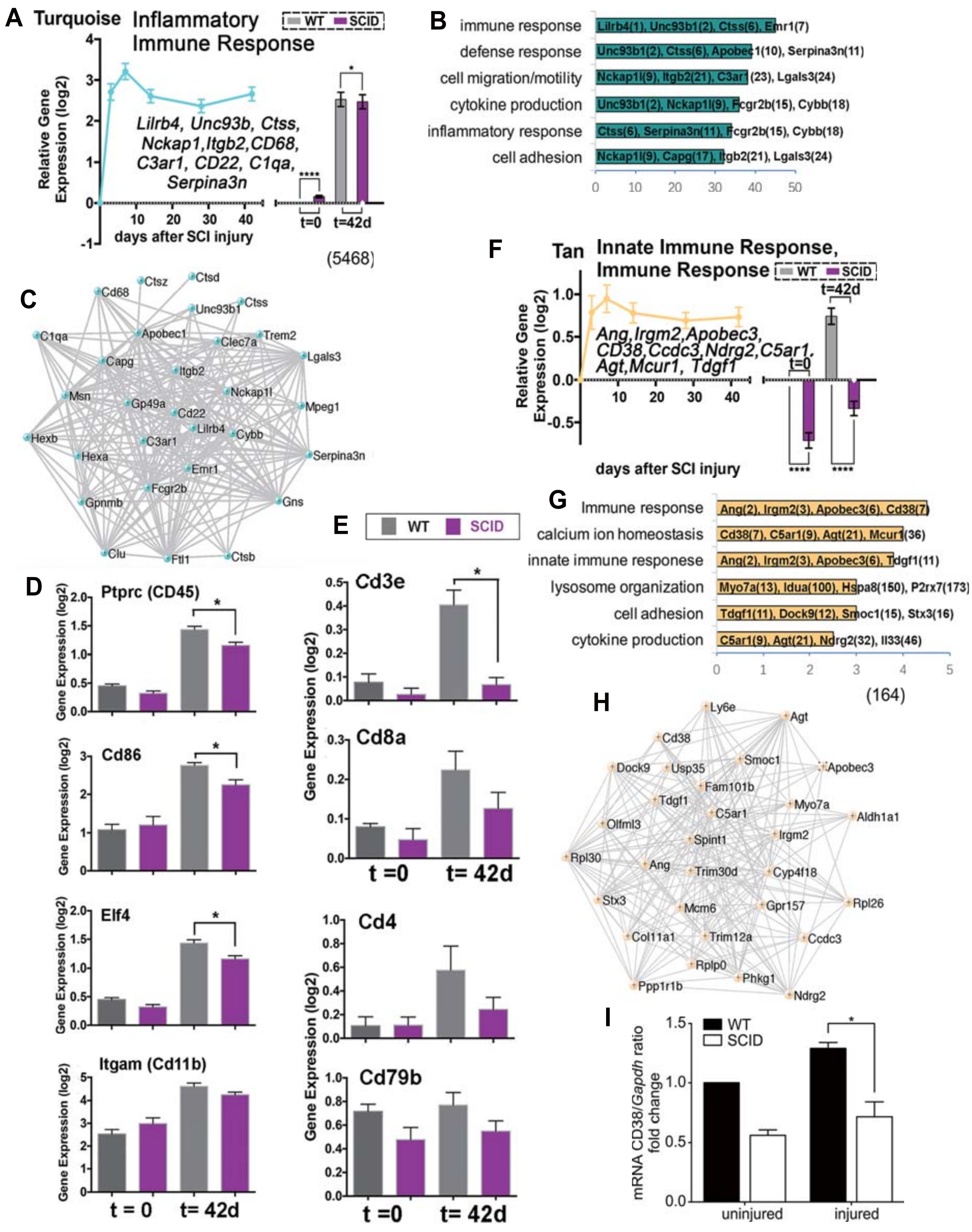

Sup. Fig 2 Luo *et al.*

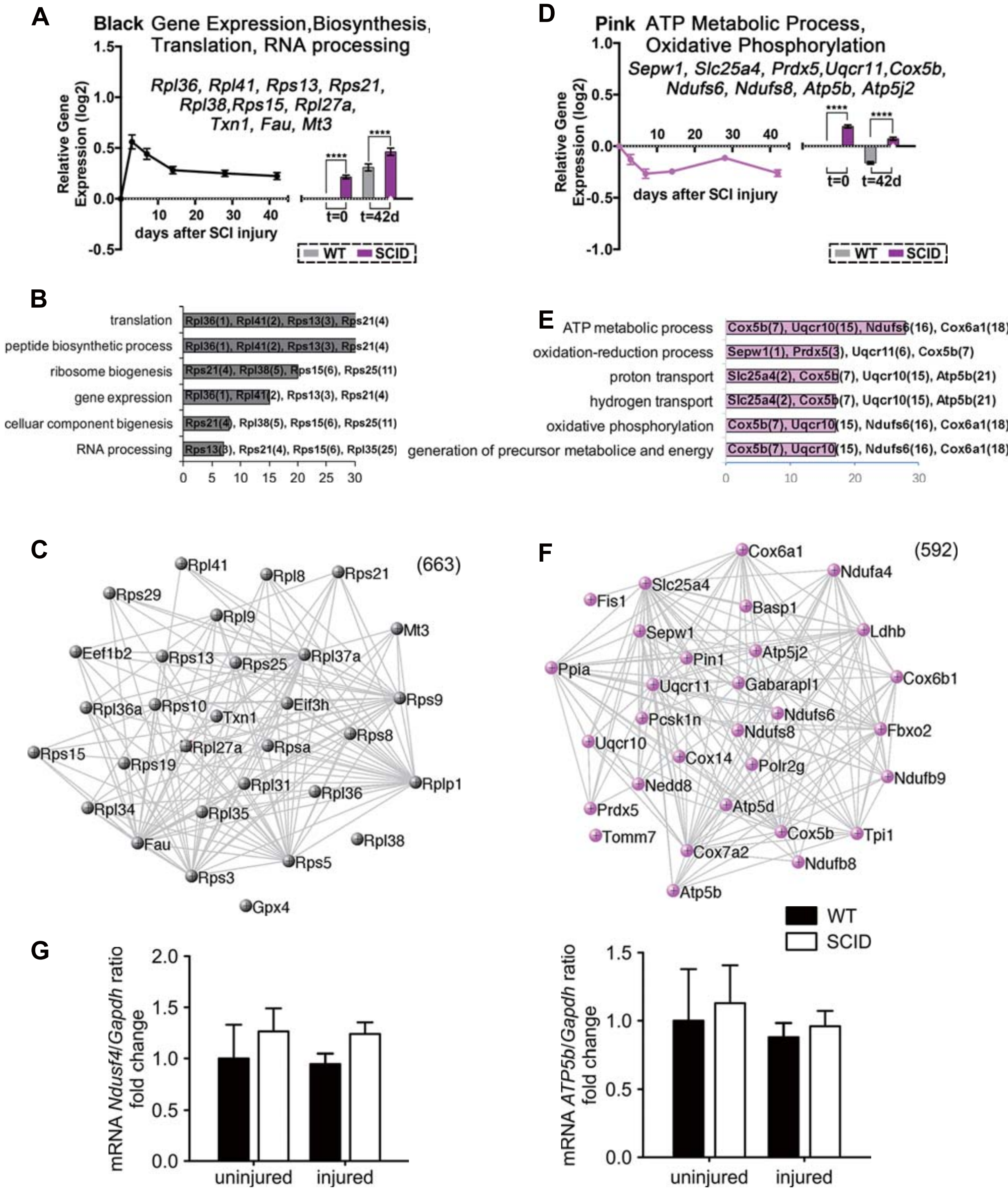

Sup. Fig 3 Luo *et al.*

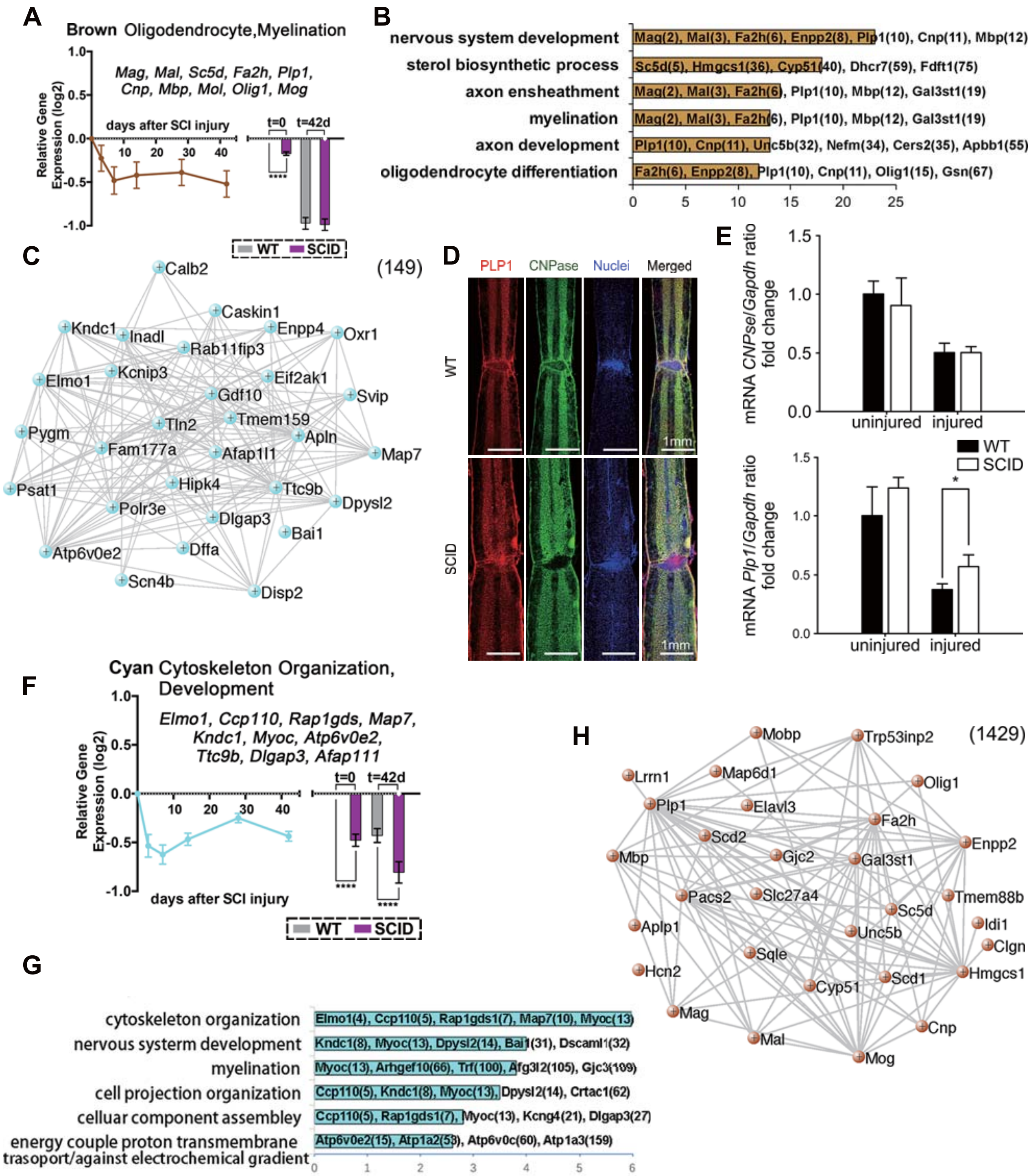

Sup. Fig 4 Luo *et al.*

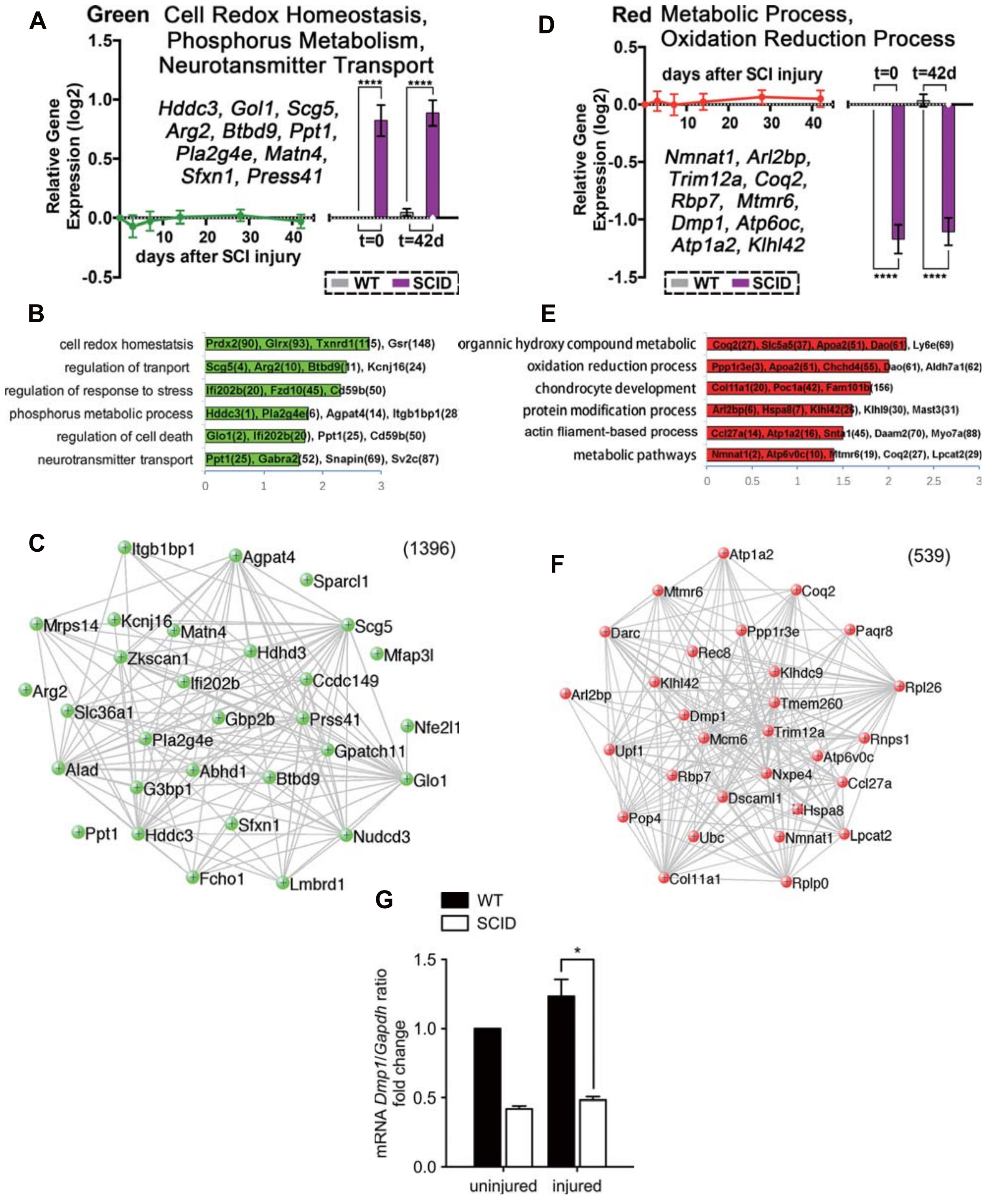

Sup. Fig 5 Luo *et al.*

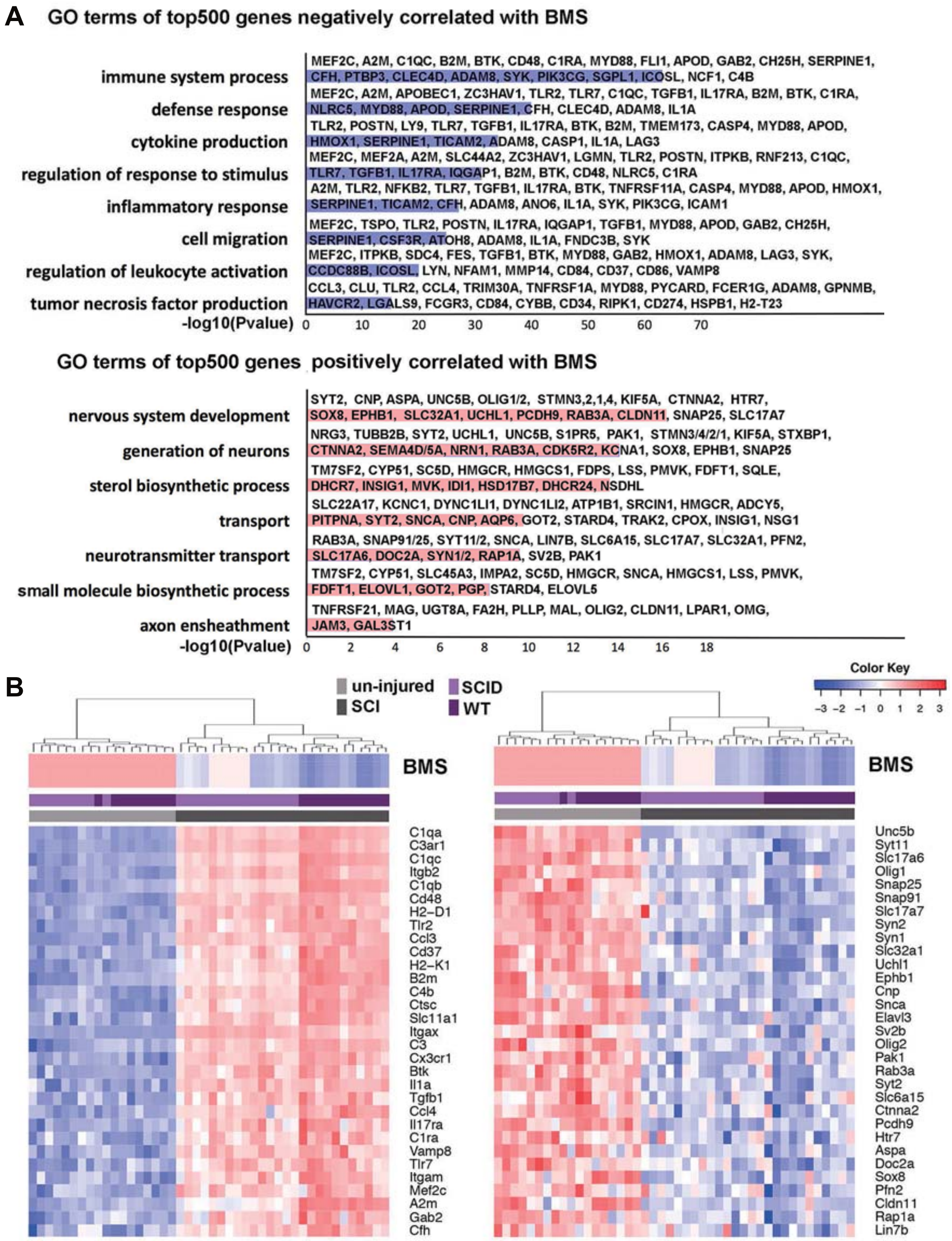

Sup Fig. 6 Luo *et al.*
